# Supplementary material for: The Effects of Genotype × Environment on Physicochemical and Sensory Properties and Differences of Volatile Organic Compounds of Three Rice Types (Oryza sativa L.)
Source: Foods. 2023 Aug 18;12(16):3108. doi: 10.3390/foods12163108 (PMC10453673; doi:10.3390/foods12163108)
Supplement: Supplementary file 1 [file foods-12-03108-s001.zip › foods-2546546-supplementary.pdf]

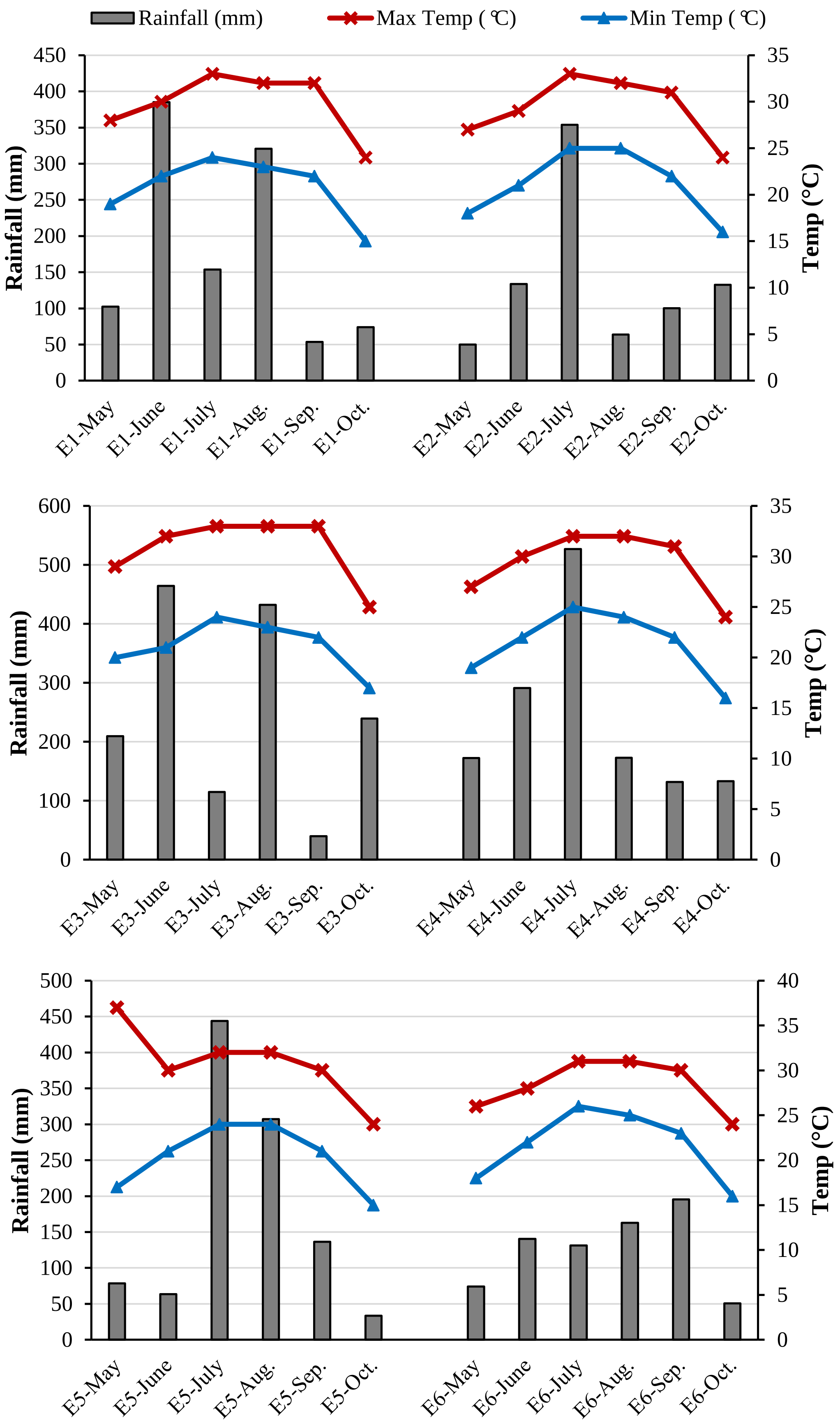

Rainfall (mm)
Max Temp (℃)
Min Temp (℃)
Rainfall (mm)
Temp (°C)
E1-May
E1-June
E1-July
E1-Aug.
E1-Sep.
E1-Oct.
E2-May
E2-June
E2-July
E2-Aug.
E2-Sep.
E2-Oct.
E3-May
E3-June
E3-July
E3-Aug.
E3-Sep.
E3-Oct.
E4-May
E4-June
E4-July
E4-Aug.
E4-Sep.
E4-Oct.
E5-May
E5-June
E5-July
E5-Aug.
E5-Sep.
E5-Oct.
E6-May
E6-June
E6-July
E6-Aug.
E6-Sep.
E6-Oct.

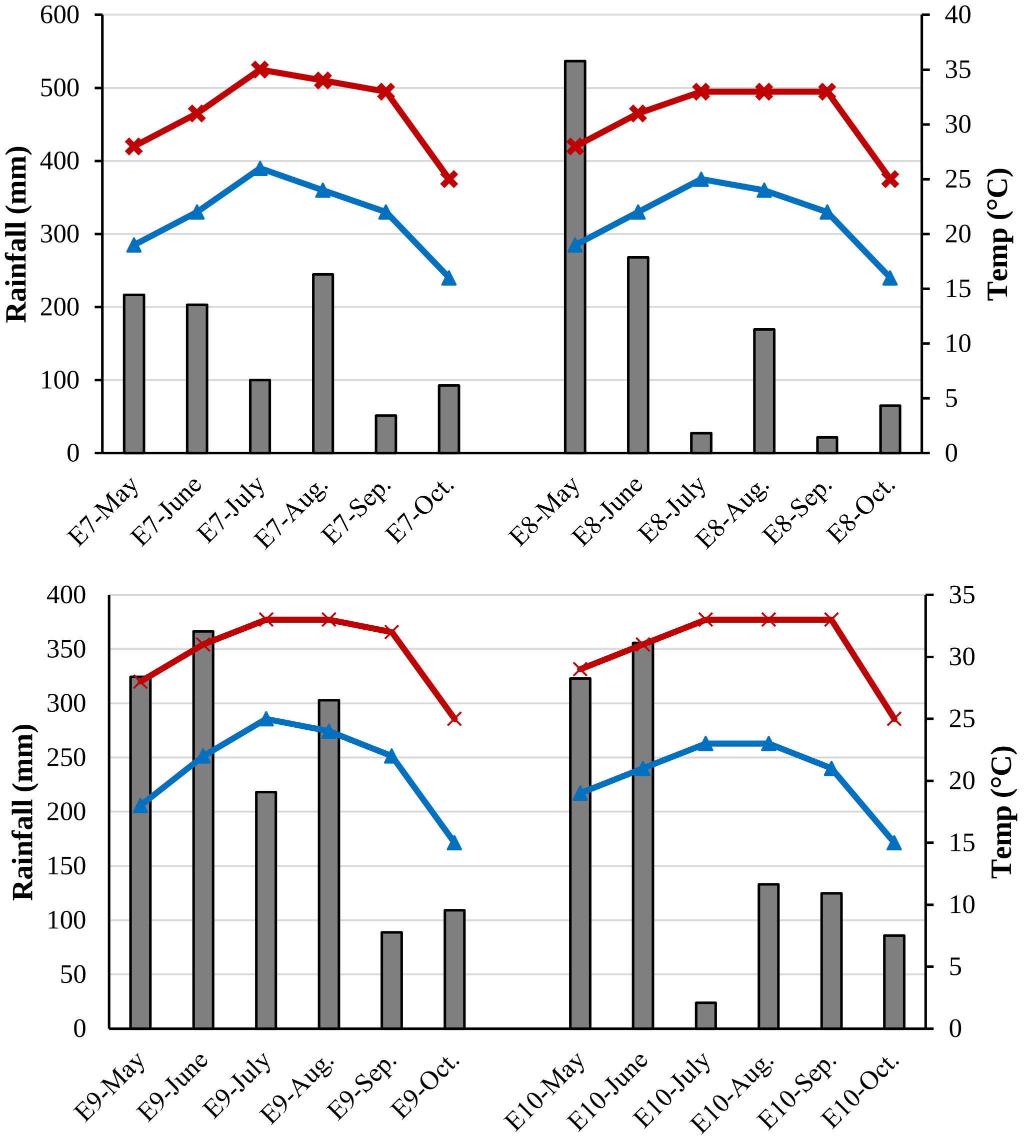

Supplementary Figure S1. Weather conditions per month during the period of rice growth in the ten environments.

Max Temp, the average of the maximum temperature per month; Min Temp, the average of the minimum temperature per month; Rainfall, total rainfall per month; E1–E10 indicates the ten environments.

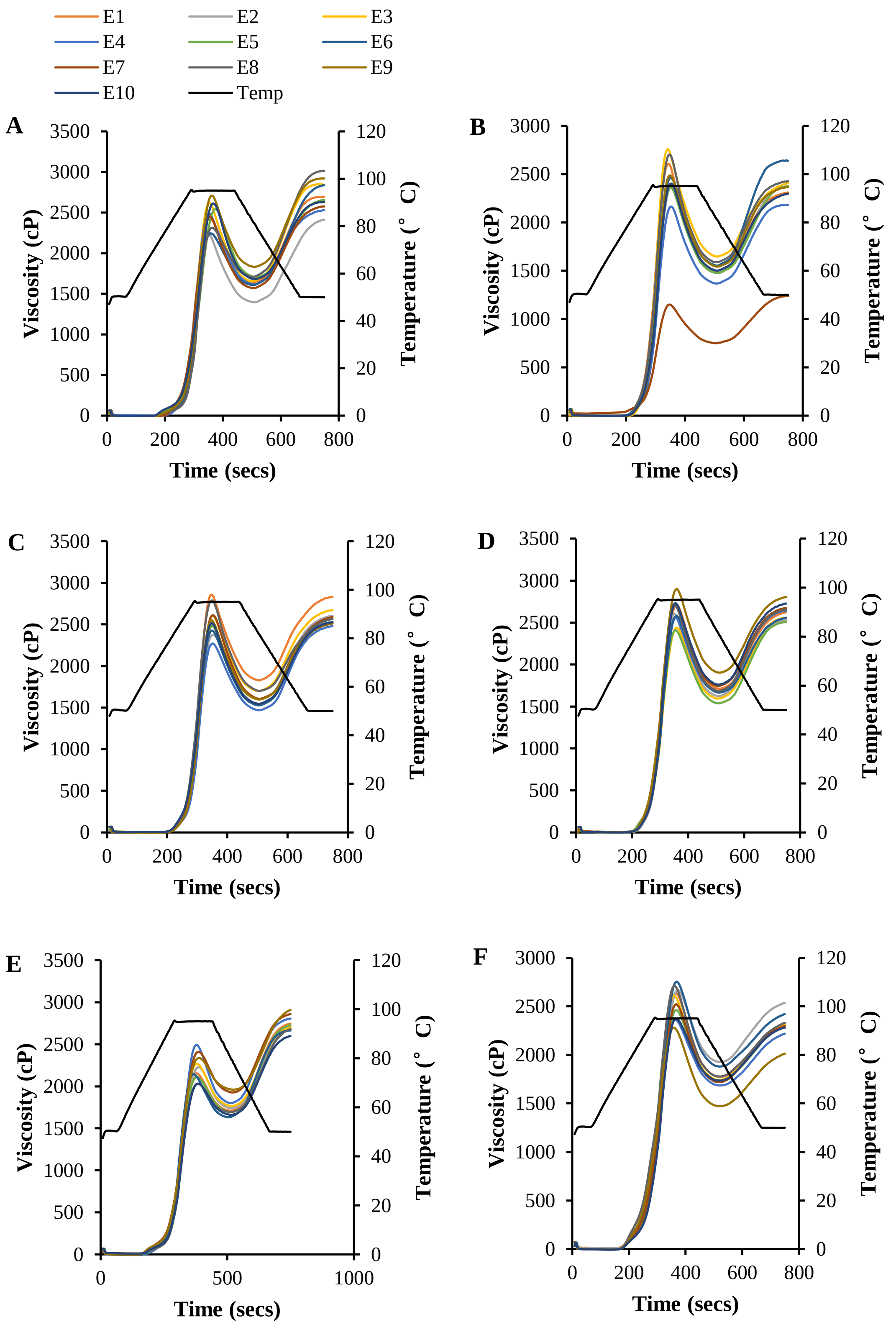

Supplementary Figure S2. The RVA profile of the six rice varieties under ten environments. A. HZY261; B. ZZY8; C. JFY2; D. YY15; E. JHX1; F. NJ46.

Supplementary Table S1. The information of the six rice varieties and ten environments [a]

Sample No.	Genotype	Environment	City	Location	Type
1	HZY261	E1	Hangzhou	119.1 °E, 29.3 °N	*Indica*
2	HZY261	E2	Ningbo	121.2 °E, 30.2 °N	*Indica*
3	HZY261	E3	Wenzhou	120.0 °E, 27.9 °N	*Indica*
4	HZY261	E4	Shaoxing	120.8 °E, 30.0 °N	*Indica*
5	HZY261	E5	Huzhou	120.3 °E, 30.6 °N	*Indica*
6	HZY261	E6	Jiaxing	120.9 °E, 30.8 °N	*Indica*
7	HZY261	E7	Jinhua	119.6 °E, 28.7 °N	*Indica*
8	HZY261	E8	Quzhou	118.6 °E, 29.0 °N	*Indica*
9	HZY261	E9	Taizhou	120.8 °E, 28.7 °N	*Indica*
10	HZY261	E10	Lishui	118.9 °E, 27.8 °N	*Indica*
11	ZZY8	E1	Hangzhou	119.1 °E, 29.3 °N	*Indica*
12	ZZY8	E2	Ningbo	121.2 °E, 30.2 °N	*Indica*
13	ZZY8	E3	Wenzhou	120.0 °E, 27.9 °N	*Indica*
14	ZZY8	E4	Shaoxing	120.8 °E, 30.0 °N	*Indica*
15	ZZY8	E5	Huzhou	120.3 °E, 30.6 °N	*Indica*
16	ZZY8	E6	Jiaxing	120.9 °E, 30.8 °N	*Indica*
17	ZZY8	E7	Jinhua	119.6 °E, 28.7 °N	*Indica*
18	ZZY8	E8	Quzhou	118.6 °E, 29.0 °N	*Indica*
19	ZZY8	E9	Taizhou	120.8 °E, 28.7 °N	*Indica*
20	ZZY8	E10	Lishui	118.9 °E, 27.8 °N	*Indica*
21	JFY2	E1	Hangzhou	119.1 °E, 29.3 °N	*Indica-japonica*
22	JFY2	E2	Ningbo	121.2 °E, 30.2 °N	*Indica-japonica*
23	JFY2	E3	Wenzhou	120.0 °E, 27.9 °N	*Indica-japonica*
24	JFY2	E4	Shaoxing	120.8 °E, 30.0 °N	*Indica-japonica*
25	JFY2	E5	Huzhou	120.3 °E, 30.6 °N	*Indica-japonica*
26	JFY2	E6	Jiaxing	120.9 °E, 30.8 °N	*Indica-japonica*
27	JFY2	E7	Jinhua	119.6 °E, 28.7 °N	*Indica-japonica*
28	JFY2	E8	Quzhou	118.6 °E, 29.0 °N	*Indica-japonica*
29	JFY2	E9	Taizhou	120.8 °E, 28.7 °N	*Indica-japonica*
30	JFY2	E10	Lishui	118.9 °E, 27.8 °N	*Indica-japonica*
31	YY15	E1	Hangzhou	119.1 °E, 29.3 °N	*Indica-japonica*
32	YY15	E2	Ningbo	121.2 °E, 30.2 °N	*Indica-japonica*
33	YY15	E3	Wenzhou	120.0 °E, 27.9 °N	*Indica-japonica*
34	YY15	E4	Shaoxing	120.8 °E, 30.0 °N	*Indica-japonica*
35	YY15	E5	Huzhou	120.3 °E, 30.6 °N	*Indica-japonica*

Sample No.	Genotype	Environment	City	Location	Type
36	YY15	E6	Jiaxing	120.9 °E, 30.8 °N	*Indica-japonica*
37	YY15	E7	Jinhua	119.6 °E, 28.7 °N	*Indica-japonica*
38	YY15	E8	Quzhou	118.6 °E, 29.0 °N	*Indica-japonica*
39	YY15	E9	Taizhou	120.8 °E, 28.7 °N	*Indica-japonica*
40	YY15	E10	Lishui	118.9 °E, 27.8 °N	*Indica-japonica*
41	JHX1	E1	Hangzhou	119.1 °E, 29.3 °N	*Japonica*
42	JHX1	E2	Ningbo	121.2 °E, 30.2 °N	*Japonica*
43	JHX1	E3	Wenzhou	120.0 °E, 27.9 °N	*Japonica*
44	JHX1	E4	Shaoxing	120.8 °E, 30.0 °N	*Japonica*
45	JHX1	E5	Huzhou	120.3 °E, 30.6 °N	*Japonica*
46	JHX1	E6	Jiaxing	120.9 °E, 30.8 °N	*Japonica*
47	JHX1	E7	Jinhua	119.6 °E, 28.7 °N	*Japonica*
48	JHX1	E8	Quzhou	118.6 °E, 29.0 °N	*Japonica*
49	JHX1	E9	Taizhou	120.8 °E, 28.7 °N	*Japonica*
50	JHX1	E10	Lishui	118.9 °E, 27.8 °N	*Japonica*
51	NJ46	E1	Hangzhou	119.1 °E, 29.3 °N	*Japonica*
52	NJ46	E2	Ningbo	121.2 °E, 30.2 °N	*Japonica*
53	NJ46	E3	Wenzhou	120.0 °E, 27.9 °N	*Japonica*
54	NJ46	E4	Shaoxing	120.8 °E, 30.0 °N	*Japonica*
55	NJ46	E5	Huzhou	120.3 °E, 30.6 °N	*Japonica*
56	NJ46	E6	Jiaxing	120.9 °E, 30.8 °N	*Japonica*
57	NJ46	E7	Jinhua	119.6 °E, 28.7 °N	*Japonica*
58	NJ46	E8	Quzhou	118.6 °E, 29.0 °N	*Japonica*
59	NJ46	E9	Taizhou	120.8 °E, 28.7 °N	*Japonica*
60	NJ46	E10	Lishui	118.9 °E, 27.8 °N	*Japonica*

a HZY261, huazheyou261; ZZY8, zhongzheyou8; JFY2, jiafengyou2; YY15, yongyou15; JHX1, jiahexiang1; NJ46, nanjing46; E1-E10, the ten environments, respectively.

Supplementary Table S2. The sensitive evaluations of the cooked rice of six rice varieties under ten environments [a]

	**FCR**	**ACR**	**PCR**	**TCR**	**TCCR**	**SEV**
Genotype						
HZY261	17.1±1.3^{A}	17.3±1.2^{A}	25.1±2.0^{A}	21.3±1.6^{A}	4.3±0.3^{A}	85.1±3.7^{A}
ZZY8	16.8±1.6^{AB}	16.5±1.3^{BC}	25.1±2.0^{A}	21.1±1.7^{AB}	4.2±0.4^{AB}	83.6±5.1^{AB}
JFY2	16.9±1.3^{AB}	16.4±1.3^{C}	24.6±1.9^{A}	20.6±1.6^{BC}	4.2±0.4^{AB}	82.7±3.8^{BC}
YY15	17.0±1.5^{AB}	16.9±1.3^{B}	24.8±2.2^{A}	20.8±1.8^{A-C}	4.2±0.3^{AB}	83.6±5.0^{AB}
JHX1	16.4±1.7^{B}	15.9±1.0^{D}	23.0±3.5^{B}	20.6±1.6^{BC}	4.0±0.4^{BC}	79.8±5.0^{D}
NJ46	16.6±1.5^{AB}	16.2±1.2^{CD}	24.4±1.9^{A}	20.3±2.0^{C}	4.0±0.4^{C}	81.6±4.2^{C}
Environment						
E1	17.3±1.2^{A}	16.5±1.3^{AB}	24.4±2.2^{A}	21.1±1.6^{A}	4.2±0.4^{A}	83.5±4.2^{AB}
E2	16.1±1.9^{B}	16.2±1.2^{B}	24.3±2.1^{A}	20.4±1.6^{A}	4.0±0.4^{A}	81.0±5.3^{B}
E3	16.7±1.4^{AB}	16.4±1.3^{AB}	24.5±1.9^{A}	20.4±1.8^{A}	4.0±0.4^{A}	82.1±4.6^{AB}
E4	16.5±1.3^{AB}	16.5±1.3^{AB}	24.0±2.0^{A}	20.8±1.7^{A}	4.2±0.4^{A}	82.0±4.6^{AB}
E5	17.0±1.3^{A}	16.4±1.3^{AB}	23.8±4.6^{A}	20.9±1.7^{A}	4.1±0.4^{A}	82.3±6.2^{AB}
E6	17.2±1.2^{A}	17.0±1.3^{A}	25.2±2.0^{A}	20.9±1.7^{A}	4.2±0.3^{A}	84.4±3.6^{A}
E7	17.2±1.3^{A}	16.1±1.2^{B}	24.7±1.9^{A}	21.0±1.7^{A}	4.2±0.3^{A}	83.1±3.5^{AB}
E8	16.7±1.5^{AB}	16.8±1.3^{AB}	24.9±2.0^{A}	20.9±1.7^{A}	4.2±0.4^{A}	83.5±4.4^{AB}
E9	16.5±2.0^{AB}	16.7±1.3^{AB}	24.4±1.9^{A}	20.2±2.0^{A}	4.1±0.5^{A}	81.9±6.0^{AB}
E10	16.9±1.7^{AB}	16.6±1.3^{AB}	24.9±2.1^{A}	21.0±1.9^{A}	4.2±0.3^{A}	83.5±5.3^{AB}

[a] The results are presented as mean ±standard deviation on 100-point scale, and values in each column of genotype or environment with different letters are significantly different ($P < 0.05$). FCR, flavor of cooked rice; ACR, appearance of cooked rice; PCR: palatability of cooked rice; TCR, taste of cooked rice; TCCR, texture of cooled cooked rice; SEV, sensitive evaluation value.

Supplementary Table S3. The compound information corresponding to the selected characteristic signals [a]

Category	Signal No.	Compounds	Rentention time (s)	Drift time (ms)	Intensity values of characteristic peacks / V																							
					HZY261-L		HZY261-H		ZZY8-L		ZZY8-H		JFY2-L		JFY2-H		YY15-L		YY15-H		JHX1-L		JHX1-H		NJ46-L		NJ46-H	
					m	sd	m	sd	m	sd	m	sd	m	sd	m	sd	m	sd	m	sd	m	sd	m	sd	m	sd	m	sd
Aldehydes	11	pentanal dimer	180.682	1.41873	0.360	0.024	0.233	0.016	0.187	0.035	0.153	0.060	0.416	0.010	0.458	0.034	0.653	0.008	0.465	0.023	0.120	0.073	0.204	0.099	0.237	0.037	0.289	0.137
	12	pentanal	180.355	1.20197	0.817	0.020	0.554	0.026	0.486	0.047	0.517	0.087	0.764	0.030	0.869	0.042	1.011	0.003	1.077	0.008	0.411	0.175	0.591	0.184	0.575	0.095	0.697	0.266
	17	phenylacetaldehyde	646.207	1.26414	0.098	0.004	0.125	0.002	0.106	0.007	0.096	0.003	0.261	0.011	0.188	0.003	0.196	0.001	0.184	0.004	0.104	0.008	0.128	0.002	0.126	0.007	0.128	0.005
	23	2-pentenal (E)	232.182	1.122	0.524	0.000	0.739	0.018	0.771	0.096	0.737	0.182	0.918	0.022	0.640	0.035	0.560	0.001	0.311	0.003	0.486	0.218	0.951	0.368	1.010	0.073	0.913	0.421
	27	2-nonenal (E)	921.024	1.40658	0.192	0.001	0.330	0.003	0.251	0.006	0.220	0.005	0.226	0.000	0.178	0.012	0.145	0.001	0.137	0.000	0.168	0.010	0.246	0.020	0.162	0.000	0.140	0.007
	28	2-furfural	303.343	1.09822	0.190	0.009	0.186	0.007	0.219	0.002	0.178	0.007	0.154	0.003	0.125	0.007	0.124	0.004	0.115	0.001	0.172	0.042	0.120	0.009	0.108	0.001	0.123	0.012
	37	(E)-2-hexenal dimer	321.762	1.51364	0.079	0.003	0.109	0.009	0.089	0.005	0.096	0.018	0.098	0.009	0.082	0.024	0.072	0.002	0.067	0.003	0.128	0.008	0.084	0.004	0.093	0.001	0.070	0.001
	38	(E)-2-hexenal	324.733	1.17923	0.280	0.002	0.372	0.001	0.326	0.007	0.345	0.009	0.404	0.016	0.325	0.004	0.349	0.003	0.324	0.003	0.427	0.021	0.353	0.009	0.380	0.021	0.262	0.002
	39	benzaldehyde dimer	511.975	1.46856	0.179	0.011	0.206	0.011	0.163	0.004	0.177	0.003	0.164	0.005	0.160	0.021	0.150	0.006	0.197	0.009	0.186	0.016	0.176	0.005	0.141	0.001	0.151	0.004
	40	benzaldehyde	512.482	1.14934	0.944	0.034	1.015	0.009	0.854	0.004	0.870	0.003	0.848	0.038	0.807	0.041	0.762	0.001	0.965	0.013	0.908	0.041	0.922	0.001	0.681	0.000	0.742	0.007
	41	butanal dimer	136.246	1.28694	0.689	0.000	0.255	0.022	0.212	0.006	0.419	0.013	0.566	0.005	0.636	0.060	0.464	0.050	0.395	0.009	0.499	0.043	0.660	0.078	0.471	0.098	0.083	0.017
	42	butanal	134.12	1.10788	2.193	0.034	2.169	0.072	2.077	0.007	2.160	0.021	2.007	0.055	1.988	0.008	1.989	0.005	2.131	0.040	2.081	0.047	1.792	0.086	1.341	0.034	0.357	0.054

	43	heptanal dimer	384.742	1.68601	0.682	0.009	0.620	0.011	0.396	0.004	0.799	0.008	0.560	0.024	0.560	0.023	0.692	0.002	0.525	0.010	0.418	0.025	0.646	0.003	0.588	0.013	0.670	0.041
	44	heptanal	385.337	1.34471	0.773	0.032	0.829	0.019	0.628	0.008	0.953	0.002	0.754	0.009	0.761	0.003	0.886	0.002	0.872	0.002	0.678	0.016	0.835	0.031	0.761	0.049	0.931	0.004
	45	hexanal dimer	260.575	1.55685	4.179	0.051	3.985	0.029	3.763	0.065	4.059	0.104	4.246	0.060	4.348	0.054	4.381	0.009	4.559	0.027	3.360	0.244	4.090	0.158	4.015	0.110	4.280	0.212
	46	hexanal	261.538	1.2685	1.517	0.013	1.452	0.009	1.430	0.024	1.528	0.033	1.499	0.035	1.547	0.019	1.546	0.008	1.680	0.021	1.304	0.068	1.495	0.044	1.441	0.030	1.537	0.055
	47	n-nonanal dimer	792.12	1.94066	1.116	0.001	1.460	0.009	1.026	0.008	1.354	0.028	0.902	0.041	0.944	0.010	0.951	0.009	1.055	0.002	1.052	0.068	1.328	0.092	0.992	0.011	1.119	0.040
	48	n-nonanal	792.12	1.48234	1.548	0.012	1.555	0.005	1.510	0.002	1.582	0.004	1.545	0.006	1.563	0.001	1.561	0.003	1.614	0.003	1.479	0.025	1.576	0.033	1.526	0.012	1.590	0.003
	49	octanal dimer	579.763	1.81432	0.144	0.000	0.157	0.002	0.101	0.003	0.225	0.010	0.189	0.004	0.203	0.000	0.291	0.004	0.191	0.001	0.162	0.009	0.245	0.003	0.251	0.003	0.264	0.004
	50	octanal	582.166	1.41562	0.624	0.025	0.711	0.001	0.582	0.001	0.856	0.006	0.795	0.013	0.819	0.034	0.970	0.011	0.804	0.024	0.711	0.008	0.896	0.010	0.903	0.020	0.922	0.013
Alcohols	1	1-pentanol dimer	242.269	1.51487	0.346	0.003	0.177	0.009	0.231	0.024	0.149	0.019	0.548	0.020	0.555	0.017	0.499	0.003	0.303	0.009	0.150	0.038	0.258	0.052	0.371	0.033	0.270	0.088
	2	1-pentanol	243.553	1.25193	1.061	0.005	0.756	0.028	0.845	0.055	0.808	0.066	1.233	0.010	1.275	0.028	1.220	0.003	1.065	0.009	0.589	0.084	0.888	0.111	1.022	0.072	0.944	0.191
	9	n-hexanol dimer	358.005	1.64464	0.441	0.022	0.250	0.007	0.386	0.012	0.183	0.004	0.764	0.003	0.703	0.028	0.559	0.015	0.388	0.020	0.321	0.036	0.383	0.022	0.648	0.012	0.354	0.055
	10	n-hexanol	359.194	1.32747	1.134	0.022	0.853	0.009	1.039	0.016	0.721	0.014	1.434	0.016	1.397	0.006	1.245	0.019	1.086	0.015	0.971	0.041	1.066	0.019	1.345	0.017	1.033	0.069
	14	3-methylbutanol	216.711	1.24153	0.407	0.012	0.264	0.006	0.342	0.035	0.345	0.055	0.447	0.001	0.507	0.019	0.535	0.003	0.441	0.009	0.303	0.123	0.309	0.072	0.322	0.051	0.351	0.118
	22	2-methyl-1-propanol	151.608	1.17613	0.485	0.004	0.320	0.017	0.287	0.003	0.469	0.009	0.635	0.010	0.684	0.028	0.784	0.005	0.396	0.015	0.621	0.008	0.579	0.024	0.867	0.011	0.607	0.036
	26	1-octanol	756.176	1.47476	0.256	0.008	0.247	0.006	0.208	0.009	0.202	0.008	0.166	0.002	0.150	0.010	0.139	0.002	0.137	0.003	0.157	0.001	0.173	0.011	0.166	0.005	0.146	0.001

	35	2-ethyl-1-hexanol dimer	661.46	1.79154	0.425	0.001	0.465	0.026	0.372	0.019	0.433	0.001	0.355	0.003	0.457	0.029	0.444	0.004	0.383	0.020	0.405	0.064	0.441	0.016	0.376	0.019	0.362	0.013
	36	2-ethyl-1-hexanol	660.002	1.4152	1.013	0.002	1.115	0.051	1.005	0.016	1.086	0.067	0.995	0.002	1.083	0.003	1.083	0.009	1.028	0.012	1.078	0.088	1.116	0.016	1.047	0.009	1.010	0.016
	51	1-butanol	164.427	1.18087	1.253	0.054	0.832	0.004	0.756	0.005	1.108	0.014	1.164	0.027	1.339	0.029	1.257	0.003	1.046	0.045	1.092	0.027	1.088	0.005	1.215	0.019	1.360	0.003
	53	1-nonanol	1046.829	1.549	0.125	0.003	0.148	0.002	0.128	0.000	0.164	0.001	0.170	0.002	0.156	0.001	0.163	0.001	0.151	0.001	0.151	0.001	0.168	0.008	0.155	0.000	0.168	0.001
	54	1-octen-3-ol	549.006	1.1707	0.846	0.057	0.778	0.018	0.730	0.014	0.841	0.020	0.828	0.016	0.881	0.048	0.839	0.005	0.855	0.017	0.930	0.009	0.960	0.013	0.774	0.007	0.873	0.014
	57	(Z)-3-hexen-1-ol	342.757	1.22843	0.085	0.005	0.066	0.007	0.064	0.003	0.146	0.016	0.101	0.008	0.132	0.006	0.114	0.002	0.125	0.003	0.099	0.004	0.137	0.001	0.129	0.011	0.142	0.008
	59	ethanol	95.915	1.04771	5.183	0.189	4.914	0.110	4.793	0.009	4.820	0.079	4.555	0.161	4.780	0.092	4.838	0.022	4.856	0.104	4.532	0.107	4.417	0.170	4.570	0.132	4.610	0.123
Ketones	3	2-heptanone dimer	368.106	1.62223	0.375	0.003	0.308	0.005	0.313	0.002	0.171	0.009	0.763	0.022	0.663	0.028	0.588	0.004	0.456	0.020	0.306	0.040	0.364	0.010	0.492	0.010	0.372	0.028
	4	2-heptanone	372.265	1.25852	0.510	0.001	0.524	0.005	0.458	0.007	0.536	0.012	0.667	0.013	0.629	0.015	0.650	0.003	0.676	0.003	0.447	0.022	0.538	0.001	0.508	0.009	0.584	0.017
	21	2-butanone	136.394	1.07139	0.244	0.009	0.092	0.002	0.082	0.014	0.156	0.038	0.195	0.009	0.243	0.019	0.314	0.010	0.245	0.001	0.133	0.002	0.294	0.002	0.533	0.031	0.502	0.096
	24	3-pentanone	176.59	1.12227	0.459	0.005	0.544	0.000	0.519	0.042	0.599	0.063	0.661	0.009	0.527	0.002	0.522	0.006	0.469	0.003	0.627	0.127	0.743	0.110	0.728	0.031	0.714	0.157
	29	acetophenone	733.866	1.19068	0.262	0.031	0.242	0.014	0.223	0.002	0.215	0.002	0.177	0.002	0.175	0.009	0.180	0.001	0.175	0.003	0.209	0.013	0.184	0.004	0.178	0.003	0.170	0.001
	52	1-hydroxy-2-propanone	158.128	1.03952	0.714	0.028	0.701	0.009	0.828	0.020	0.822	0.014	0.587	0.001	0.659	0.022	0.706	0.019	0.588	0.007	0.757	0.065	0.620	0.027	0.693	0.012	0.582	0.009
	55	2-hexanone	249.655	1.18785	0.271	0.010	0.275	0.002	0.292	0.006	0.361	0.000	0.302	0.004	0.302	0.016	0.272	0.003	0.305	0.006	0.209	0.012	0.287	0.002	0.228	0.005	0.237	0.003
	56	2-propanone	104.609	1.11966	3.894	0.084	3.408	0.047	3.059	0.022	2.864	0.081	3.790	0.085	4.120	0.054	4.252	0.031	4.010	0.025	3.743	0.043	4.096	0.107	4.094	0.060	4.511	0.069

	58	cyclohexanone	383.554	1.15338	0.121	0.005	0.144	0.002	0.125	0.007	0.153	0.009	0.109	0.009	0.103	0.004	0.120	0.007	0.173	0.001	0.162	0.005	0.143	0.008	0.116	0.005	0.159	0.013
Esters	15	ethyl propanoate	189.387	1.15331	0.151	0.004	0.161	0.009	0.170	0.024	0.195	0.028	0.216	0.005	0.238	0.012	0.220	0.002	0.305	0.001	0.157	0.046	0.168	0.033	0.153	0.015	0.167	0.029
	16	methyl hexanoate	423.261	1.28051	0.129	0.000	0.107	0.003	0.093	0.005	0.139	0.005	0.160	0.005	0.173	0.008	0.183	0.000	0.172	0.003	0.131	0.009	0.133	0.003	0.129	0.003	0.142	0.006
	25	ethyl acrylate	193.967	1.12149	0.837	0.007	1.155	0.060	1.032	0.157	1.125	0.265	1.351	0.020	1.053	0.039	0.961	0.042	0.723	0.003	1.198	0.184	1.292	0.331	1.474	0.136	1.281	0.275
	30	ethyl acetate	141.825	1.33592	5.745	0.105	6.438	0.003	6.477	0.011	5.809	0.004	4.914	0.008	4.087	0.132	2.916	0.218	4.091	0.100	5.585	0.024	3.183	0.127	1.050	0.013	0.108	0.006
	31	propyl hexanoate	809.473	1.38575	0.175	0.013	0.190	0.012	0.173	0.004	0.159	0.013	0.159	0.009	0.140	0.005	0.137	0.002	0.127	0.003	0.118	0.003	0.132	0.004	0.139	0.016	0.125	0.001
Alkenes	5	2-heptenal (E) dimer	481.726	1.66054	0.475	0.156	0.233	0.001	0.176	0.003	0.167	0.001	0.351	0.020	0.260	0.005	0.298	0.001	0.268	0.015	0.230	0.049	0.193	0.005	0.197	0.003	0.179	0.039
	6	2-heptenal (E)	487.012	1.25471	0.533	0.000	0.723	0.000	0.618	0.006	0.620	0.007	0.908	0.006	0.773	0.029	0.848	0.006	0.805	0.011	0.669	0.024	0.671	0.007	0.671	0.007	0.616	0.006
	7	2-octenal (E) dimer	700.494	1.80796	0.099	0.004	0.146	0.001	0.103	0.003	0.109	0.004	0.212	0.017	0.189	0.012	0.197	0.003	0.159	0.011	0.121	0.024	0.145	0.003	0.131	0.005	0.115	0.010
	8	2-octenal (E)	700.57	1.33088	0.328	0.006	0.401	0.004	0.350	0.001	0.308	0.002	0.620	0.015	0.499	0.014	0.504	0.008	0.479	0.002	0.316	0.022	0.364	0.016	0.392	0.020	0.372	0.019
Pyrroles	20	2-acetyl-1-pyrroline	429.229	1.12693	0.321	0.035	0.349	0.012	0.331	0.023	0.475	0.036	0.326	0.199	0.190	0.009	0.122	0.012	0.087	0.004	1.086	0.070	1.005	0.051	0.922	0.016	1.133	0.030
Furans	13	2-pentylfuran	550.972	1.24982	0.494	0.017	0.463	0.004	0.443	0.000	0.309	0.003	0.736	0.021	0.595	0.009	0.625	0.007	0.463	0.017	0.286	0.016	0.404	0.007	0.530	0.006	0.378	0.020
Undefied	18	ID_1	540.356	1.43698	0.110	0.003	0.085	0.001	0.052	0.000	0.110	0.004	0.161	0.009	0.123	0.009	0.173	0.002	0.118	0.001	0.087	0.004	0.115	0.003	0.121	0.003	0.123	0.007
	19	ID_2	550.561	1.67688	0.067	0.001	0.068	0.003	0.068	0.001	0.051	0.004	0.121	0.003	0.119	0.035	0.142	0.004	0.132	0.004	0.049	0.003	0.069	0.003	0.070	0.004	0.063	0.003
	32	ID_3	134.386	1.19541	0.343	0.007	0.355	0.043	0.328	0.024	0.350	0.025	0.264	0.003	0.236	0.003	0.207	0.004	0.259	0.002	0.298	0.018	0.209	0.002	0.142	0.015	0.146	0.016

	33	ID_4	116.584	1.1946	0.157	0.008	0.309	0.023	0.513	0.012	0.134	0.001	0.149	0.019	0.143	0.014	0.188	0.002	0.088	0.019	0.140	0.008	0.172	0.014	0.166	0.040	0.164	0.021
	34	ID_5	112.333	1.15686	0.345	0.007	0.669	0.019	0.872	0.003	0.479	0.095	0.176	0.008	0.181	0.009	0.242	0.018	0.111	0.000	0.451	0.082	0.225	0.003	0.242	0.148	0.123	0.005

[a] HZY261-L and HZY261-H, Huazheyou261 with the lowest and highest flavor of cooked rice in sensory evaluation values, respectively; ZZY8-L and ZZY8-H, Zhongzheyou8 with the lowest and highest flavor of cooked rice in sensory evaluation values, respectively; JFY2-L and JFY2-H, Jiafengyou2 with the lowest and highest flavor of cooked rice in sensory evaluation values, respectively; YY15-L and YY15-H, Yongyou15 with the lowest and highest flavor of cooked rice in sensory evaluation values, respectively; JHX1-L and JHX1-H, Jiahexiang1 with the lowest and highest flavor of cooked rice in sensory evaluation values, respectively; NJ46-L and NJ46-H, Nanjing46 with the lowest and highest flavor of cooked rice in sensory evaluation values, respectively.
